# Phytochemicals and Antioxidative Properties of Borneo Indigenous Liposu (*Baccaurea lanceolata*) and Tampoi (*Baccaurea macrocarpa*) Fruits

**DOI:** 10.3390/antiox3030516

**Published:** 2014-07-30

**Authors:** Mohd Fadzelly Abu Bakar, Nor Ezani Ahmad, Fifilyana Abdul Karim, Syazlina Saib

**Affiliations:** 1 Institute for Tropical Biology and Conservation, Universiti Malaysia Sabah, Jalan UMS, Kota Kinabalu, Sabah 88400, Malaysia; E-Mails: misznarney@gmail.com (N.E.A.); fifilyana1111@gmail.com (F.A.K.); syazlina1991@gmail.com (S.S.); 2 Faculty of Science, Technology and Human Development, Universiti Tun Hussein Onn Malaysia (UTHM), Parit Raja, Batu Pahat, Johor 86400, Malaysia

**Keywords:** *Baccaurea lanceolata*, *Baccaurea macrocarpa*, phenolic, flavonoid, anthocyanin, carotenoid, antioxidant activity

## Abstract

Two underutilized indigenous fruits of Borneo, Liposu (*Baccaurea lanceolata*) and Tampoi (*Baccaurea macrocarpa*) were investigated for their total phenolic (TPC), flavonoid (TFC), anthocyanin (TAC) and carotenoid (TCC) contents as well as antioxidant properties *in vitro*. The fruits were separated into three different parts (*i.e.*, pericarp, flesh and seed) and extracted using 80% methanol. Antioxidant activity was determined using DPPH (2,2-diphenyl-1-picrylhydrazyl) free radical scavenging, ABTS decolorization and FRAP (Ferric Reducing Antioxidant Power) assays. The results showed that *B. macrocarpa* pericarp contained the highest amount of total phenolics, total flavonoid, total anthocyanin and total carotenoid with the values of 60.04 ± 0.53 mg GAE/g, 44.68 ± 0.67 mg CE/g, 1.23 ± 0.20 mg c-3-gE/100 g and 0.81 ± 0.14 mg BCE/g. Results from DPPH, ABTS and FRAP assays also showed that the pericarp of *B. macrocarpa* displayed the highest antioxidant capacity. The antioxidant activity of the extract was significantly correlated with the total phenolic and flavonoid contents, but not with the carotenoid contents. In conclusion, *B. macrocarpa* displayed high potential as natural source of phytochemicals with antioxidant properties.

## 1. Introduction

Exogenous antioxidants are essential in the human body due to depletion of natural antioxidants [1]. They prevent or delay oxidation when exposed to free radicals and reactive oxygen species which are generated continuously inside the human body [2]. Reactive oxygen species might cause oxidative damage that leads to chronic diseases such as cancer, neurodegenerative disease, arthritis and diabetes mellitus [3]. Niki [4] reported that antioxidants can be evaluated by many methods but often give inconclusive and conflicting results. Therefore, assessment of antioxidants using more than one method is suggested to give more consistent results [5].

Previous studies have reported that high consumption of fresh fruits and vegetables may have protective effects against a wide range of chronic diseases caused by oxidative stress in the body [6]. This is mainly due to the presence of antioxidants constituents and bioactive compounds such as vitamin C, carotenoid, phenolics, flavonoids, tannins and anthocyanidins that are able to scavenge free radicals and inhibit lipid peroxidation [7,8,9]. A comprehensive analysis conducted based on the epidemiological studies available in the literature shows that there is undoubted evidence that higher consumption of fruits and vegetables reduces the risk of cardiovascular disease as well as cancer [10,11,12].

Despite many research on commonly consumed fruits, little information is available for underutilized indigenous fruits. Underutilized fruit is defined as fruit that is less popular, with under-exploited potential and not ready for commercialization [13]. Borneo (Malaysia—Sabah and Sarawak; Indonesia—Kalimantan and Brunei) has more than hundred types of indigenous fruits that can be found in backyards, small-scale orchards and also tropical rainforests. However, many of the indigenous fruits in Borneo are considered underutilized since they are only available locally and not much research has been conducted on these fruits. These underutilized fruits may contain a significant amount of phytochemicals or other unique compound which might have health-promoting properties. Their antioxidant capacity may be comparable or even superior to that of the more extensively studied fruits [14].

*Baccaurea macrocarpa* and *Baccaurea lanceolata* come from the genera of *Baccaurea* (family: Euphorbiaceae). Most of the *Baccaurea* species are endemic to Borneo. Both *B. macrocarpa* and *B. lanceolata* fruits are green to purple when young, and turn into yellow to orange when ripe. Both species are also dioecious, having the male and female reproductive organs borne on separate individuals of the same species, and can be propagate by seed. Although both species almost resembles each other, unlike *B. lanceolata*, *B. macrocarpa* trees have buttresses. *B. lanceolata* usually have one to four seeded berries with globose to ellipsoid fruit shape while the *B. macrocarpa* only have two to three seed berries with oblongoid to globose fruit shape [15]. For *B. lanceolata*, the pericarp and flesh are edible while its seed is discarded. Meanwhile, only the flesh of *B. macrocarpa* is edible while the other part, pericarp and seed are discarded [16]. *B. lanceolata* bears thick skin, white arils, with a tasty, sour flavor which is common in the wild and unknown in cultivation while the *B. macrocarpa* have tangy sweet taste, white arils around the seeds is uncommon in the wild and already introduced in garden cultivation [17].

Plants of *Baccaurea* species have been shown to contain diverse phytochemicals and pharmacological properties [16,18,19,20]. The present study was conducted to investigate the potential of *B. lanceolata* and *B. macrocarpa* fruits as a new source of natural bioactive phytochemicals and nutraceutical.

## 2. Materials and Methods

### 2.1. Plant Material and Sample Preparation

The whole fruits of *B. macrocarpa* and *B. lanceolata* were collected from Beaufort, Sabah, Malaysia. The fruits were cleaned and separated into pericarp, flesh and seed. Authentication of the fruits was done by Mr Johnny Gisil from Institute for Tropical Biology and Conservation of Universiti Malaysia Sabah, Malaysia. The pericarp, flesh and seed of the fruits were freeze-dried and ground to a fine powder. The samples were then kept in air-tight container and stored in freezer with temperature of −20 °C.

### 2.2. Extraction

All samples were extracted using 80% methanol (at a ratio of 1:20) at room temperature on an orbital shaker for 12 h at 200 rpm [21]. The resulting slurry was filtered through a Whatman No. 1 filter paper and subsequently used for determination of total phenolic, flavonoid, anthocyanin and carotenoid contents as well as total antioxidant activity.

### 2.3. Determination of Total Phenolic Content (TPC)

Total phenolic content was determined spectrophotometrically using Folin-Ciocalteu’s reagent as described by Velioglu *et al.* [21]. One hundred micro liters of sample extract was mixed with 750 μL of Folin-Ciocalteu reagent solution. The solution was mixed well using vortex and then allowed to stand at room temperature for 5 min. About 750 μL of sodium bicarbonate solution was then added to the mixture. The solution again allowed to stand at room temperature for 90 min. After 90 min, absorbance was measured at 725 nm using spectrophotometer. Gallic acid was used as standard. Total phenolic contents of the extracts were determined from the standard graph. The results were expressed as mg gallic acid equivalents in 1 g of sample (mg GAE/g).

### 2.4. Determination of Total Flavonoid Content (TFC)

Total flavonoid content was determined using aluminum chloride spectrophotometry assay as described by Zhishen *et al.* [22]. About 1 mL of sample extract was added to a beaker. Four (4) mL of distilled water were then added to the mixture followed by 0.3 mL of 5% sodium nitrite. After 6 min, 0.6 mL 10% AlCl_3_ was added and allowed to stand for 5 min. Then, 2 mL 1M NaOH and 2.1 mL of distilled water were added to the mixture and mixed using vortex. Absorbance of the mixture was determined at 510 nm. Catechin was used as standard. The total flavonoid contents of the extracts were determined from the standard graph. The total flavonoid content of the extracts was expressed as mg catechin equivalents in 1 g of sample (mg CE/g).

### 2.5. Determination of Total Anthocyanin Content (TAC)

The total anthocyanin content was determined by the pH-differential method as described by Giusti and Wrolstad [23] where the anthocyanin pigments undergo reversible structural transformations with a change in pH manifested by strikingly different absorbance spectra, which permits accurate and rapid measurement of the total anthocyanins. The colored oxonium form predominates at pH 1.0 and the colorless hemiketal form at pH 4.5. 0.5 mL of sample extract was mixed with 3.5 mL of 0.025 M potassium chloride buffer pH 1. The mixture were then allowed to mix properly using a vortex and allowed to stand for 15 min. After 15 min, absorbance at 515 and 700 nm was measured using spectrophotometer. The same mixture was then combined with 3.5 mL of 0.025 M sodium acetate buffer pH 4.5 and allowed to stand for 15 min. After 15 min, absorbance again was measured at 515 and 700 nm using spectrophotometer. The total anthocyanin content was expressed as mg of cyanidin-3-glucoside equivalents in 100 g of dried sample (mg c-3-gE/100 g dried sample) and was calculated using the formula as follows:

The total anthocyanin content (mg/100 g of dried sample) = A × MW × DF × 1000/(e × C)
(1)

where A is absorbance = (A515 − A700)_pH1.0_ − (A515 − A700)_pH4.5_; MW is a molecular weight + cyanidin-3-glucoside = 449.2; DF is the dilution factor of the samples, e is the molar absorbity of cyanidin-3-glucoside = 26,900; and C is the concentration of the buffer in mg/mL.

### 2.6. Determination of Total Carotenoid Content (TCC)

Total carotenoid content was determined according to Hess *et al.* [24]. Three hundred micro liters of sample extract was added to 300 μL of distilled water and 600 μL of 80% methanol in a test tube. The mixture was then mixed with 1200 μL of *n*-hexane solution. The final solution was then centrifuged at 2000 rpm at 4 °C for 5 min. After that, the solution should form two layers of solvents. The upper layer (contained carotenoid) was transferred to a cuvette and the absorbance was measured at 350 nm. The results were expressed as mg beta carotene equivalents in 1 g of sample (mg BCE/g).

### 2.7. Antioxidant Assessment

#### 2.7.1. DPPH (2,2-Diphenyl-1-pycryl-hydrazyl) Free Radical Scavenging Assay

The free radical scavenging ability of the extracts was determined according to Mensor *et al.* [25]. One mililiter of 0.3 mM DPPH solution was added to a test tube. Then, 2.5 mL of sample extracts or standards was added to the mixture. The mixture was then allowed to stand for 30 min in room temperature. The mixture was transferred to a cuvette and the absorbance value was then measured using spectrophotometer at 518 nm. The Antioxidant Activity (AA) was calculated as:

AA% = 100 − [(Abs sample − Abs empty sample)/(Abs control)] × 100
(2)

where Abs is absorbance.

The result obtained is expressed as ascorbic acid equivalent antioxidant capacity (AEAC) in 1 g of dry sample using the following equations:

AEAC = (IC_50 (AA)_/IC_50(sample)_) × 10^5^ (3)

where AA is ascorbic acid.

#### 2.7.2. ABTS Decolourization Assay

This assay was conducted by using ABTS as a model of free radical [26]. Briefly, an equal amount of 7 mM ABTS solution and 2.45 mM potassium persulfate was mixed and allowed to stand for 15 h in the dark under room temperature. Later, the solution was diluted with methanol to obtain absorbance of 0.7 ± 0.2 units at 734 nm. The plants extracts were separately dissolved in methanol to yield a concentration of 1 mg/mL. About 200 μL of methanolic test solution of each sample was added to 2 mL of ABTS free radical cation solution. The solution was allowed to mix well using vortex for 45 s. The solution was then transferred into a cuvette and measured at 734 nm. The result obtained was expressed as the ascorbic acid equivalent antioxidant capacity (AEAC) in 1 g of dry sample.

#### 2.7.3. FRAP (Ferric Reducing/Antioxidant Power) Assay

This procedure was done according to Benzie and Strain [27] with slight modification. The working FRAP reagent was produced by mixing 20 mL of TPTZ, 200 mL acetate buffer, and 20 mL ferric chloride and 24 mL distilled water in a 10:1:1 ratio prior to use and heated to 37 °C in a water bath. A total of 0.3 mL FRAP reagent was added to a cuvette and blank reading was then taken at 593 nm using spectrophotometer. Then, a total of 100 μL of sample extracts and 300 μL distilled water was then transferred to a cuvette and measured at 593 nm using spectrophotometer. The sample extracts were then added to the FRAP reagent. After 4 min, second reading at 593 nm was performed. The change in absorbance after 4 min from the initial blank reading was then compared with the standard curve. A standard of known Fe(II) concentration were run using several concentrations from 100 to 1000 μM. A standard curve was prepared by plotting the FRAP value of each standard *versus* its concentration. The final result was expressed as the concentration of antioxidant having ferric reducing ability.

### 2.8. Statistical Analysis

All experiments were carried out in triplicates and results are expressed as average of three independent experiments (mean ± standard deviation). Correlation analysis between antioxidant activity and phytochemical contents was analyzed using Pearson’s correlation in SPSS version 11.0 program (SPSS Inc., Chicago, IL, USA).

## 3. Results and Discussion

Determination of total phenolic (TPC), flavonoid (TFC), anthocyanin (TAC) and carotenoid (TCC) contents of pericarp, flesh and seed extracts of *B. lanceolata* and *B. macrocarpa* were determined spectrophotometrically using standard methods. TPC of the fruits extracts ranged from 3.29 to 4.81 mg GAE/g for *B. lanceolata*, while it ranged from 2.74 to 60.04 mg GAE/g for *B. macrocarpa* (Table 1). Pericarp of *B. macrocarpa* contained the highest TPC while the lowest TPC was observed in the seed of *B. macrocarpa*. The same trend of TFC was observed in *B. macrocarpa* extracts. This study was supported by Khir Jauhari *et al.* [18] who reported that peel of Belimbing Dayak (*B. angulata*) contained higher TPC and TFC as compared to the other parts of the fruits.

**Table 1:** Phytochemicals contents of *B. lanceolata* and *B. macrocarpa*.

Samples	TPC ^a^	TFC ^b^	TAC ^c^	TCC ^d^
*B. lanceolata*				
**Pericarp**	3.31 ± 0.48	2.29 ± 0.01	0.50 ± 0.13	0.75 ± 0.00
**Flesh**	4.81 ± 0.14	4.73 ± 0.27	0.37 ± 0.08	0.67 ± 0.15
**Seed**	3.29 ± 0.33	1.97 ± 0.19	0.07 ± 0.00	0.64 ± 0.28
*B. macrocarpa*				
**Pericarp**	60.04 ± 0.53	44.68 ± 0.67	1.23 ± 0.20	0.81 ± 0.14
**Flesh**	4.60 ± 0.10	1.51 ± 0.11	0.01 ± 0.01	0.69 ± 0.22
**Seed**	2.74 ± 0.24	2.14 ± 0.17	0.04 ± 0.00	0.47 ± 0.17

^a^ TPC was expressed as mg gallic acid equivalent (mg GAE) in 1 g of dry sample; ^b^ TFC was expressed as mg catechin equivalent (mg CE) in 1 g of dry sample; ^c^ TAC was expressed as mg of cyanidin-3-glucoside equivalents (mg c-3-gE) in 100 g of dry sample; ^d^ TCC was expressed as mg beta carotene equivalents (mg BCE) in 1 g of dry sample.

Meanwhile, the amount of total anthocyanin content was relatively low in all samples tested (Table 1). In *B. lanceolata*, anthocyanin content ranged from 0.07 to 0.50 mg c-3-gE/100g. For *B. macrocarpa*, TAC were in the ranged only from 0.01 to 1.23 mg c-3-gE/100g. Pericarp of *B. macrocarpa* contained the highest TAC while the flesh of *B. macrocarpa* displayed the lowest TAC. According to Vangdal and Slimestad [28], TAC usually higher in dark-red color fruit (*i.e.*, cherry) as compared to pale-yellow color fruit (*i.e.*, plum).

On the other hand, a very low total carotenoid content was observed in all sample extracts. The TCC of the samples were in the ranged of 0.47 to 0.81 mg BCE/g (Table 1). Pericarp of *B. macrocarpa* contained the highest amount of TCC with the value of 0.81 mg BCE/g while seed of *B. macrocarpa* contained the lowest TCC with the value of 0.47 mg BCE/g.

Three methods (DPPH, ABTS and FRAP) were used for antioxidant estimation. In DPPH assay, extract of *B. macrocarpa* (pericarp) showed the highest scavenging effects; followed by the flesh of *B. macrocarpa* and the flesh of *B. lanceolata* (Table 2). In ABTS assay, the antioxidant capacity ranged from 2.16 to 9.97 mg/g; where *B. macrocarpa* (pericarp) displayed the highest scavenging activity, while pericarp of *B. lanceolata* displayed the lowest scavenging effect in this assay. In FRAP assay, the pericarp of *B. macrocarpa* displayed superior antioxidant (reducing effects) as compared to other samples.

Correlation analysis revealed that the DPPH assay was moderately correlated with the total phenolic (*r*^2^ = 0.794, *p* < 0.05) and the flavonoid content (*r*^2^ = 0.796, *p* < 0.05). Previous studies on the antioxidant activity of nectarines and peach showed a strong correlation between phenolic content and antioxidant capacity [29]. However, no correlation was observed between DPPH with TAC and TCC. These results were supported by previous literature which reported that anthocyanins could be easily degraded under different conditions (*i.e.*, high temperature and humidity) [14,29,30].

**Table 2:** Antioxidant activity of *B. lanceolata* and *B. macrocarpa*.

Samples	DPPH ^a^	ABTS ^b^	FRAP ^c^
*B. lanceolata*			
**Pericarp**	48.93 ± 0.02	2.16 ± 0.11	2.29 ± 0.12
**Flesh**	94.36 ± 0.02	2.99 ± 0.12	2.81 ± 0.23
**Seed**	85.06 ± 3.15	3.03 ± 0.11	1.93 ± 0.17
*B. macrocarpa*			
**Pericarp**	196.94 ± 0.43	9.97 ± 0.30	102.88 ± 0.57
**Flesh**	97.35 ± 0.21	3.04 ± 0.11	1.90 ± 0.12
**Seed**	42.99 ± 0.17	2.90 ± 0.09	2.31 ± 0.15

^a^ DPPH free radical scavenging was expressed as mg ascorbic acid equivalent antioxidant capacity (AEAC) in 1 g of dry sample; ^b^ ABTS decolorization assay was expressed as mg ascorbic acid equivalent antioxidant capacity (AEAC) in 1 g of dry sample; ^c^ FRAP was expressed as mM ferric reduction to ferrous in 1 g of dry sample.

Unlike DPPH, analysis of correlation revealed that the ABTS was strongly correlated with the total phenolic (*r*^2^ = 0.994, *p* < 0.01) and the flavonoid content (*r*^2^ = 0.992, *p* < 0.01). The same trend of correlation was also found by Gorenstein *et al.* [31] whereby the correlation analyzed between the polyphenols and antioxidant activity as assessed by two different methods showed that phenolics and flavonoid may contribute maximally to the ABTS assay and to a lesser extent to DPPH assay. There is also strong positive correlation between ABTS and TAC (*r*^2^ = 0.862, *p* < 0.05). However, there is no correlation between ABTS and TCC (*p* > 0.05).

A strong positive correlation (*r*^2^ = 0.999, *p* < 0.01) between TPC and reducing power of extracts suggested that phenolic compounds in *Baccaurea* species might have acted as powerful reducing agent. Li *et al.* [32] reported the existence of similar linear relationships between reducing power and TPC. Similarly, a strong correlation (*r*^2^ = 0.998, *p* < 0.01) was determined between TFC and FRAP assay. This result was supported by Gil *et al.* [29] and Sabli *et al.* [33]. Similarly with ABTS assay, there is a strong correlation between TAC and FRAP assay, despite their low concentration in both *Baccaurea* fruits (*r*^2^ = 0.907, *p* < 0.05). However, TCC was not correlate with FRAP and DPPH assays.

Based on the results obtained, the edible part of *B. lanceolata* has the highest phytochemical content as compared to non-edible parts whereas the flesh of *B. macrocarpa* contained considerable amount of TPC and TFC. The pericarp and seed of *B. macrocarpa* showed the highest phytochemical contents compared to the flesh part. The consumption of edible part of these fruits was considered safer and suitable for human direct consumption as it may reduce the risk of chronic diseases that are caused by the oxidation of free radicals and reactive oxygen species.

## 4. Conclusions

In conclusion, the pericarp of *B. macrocarpa* has the highest phytochemicals and displayed strong antioxidant activity *in vitro*. The results of this study also suggested that non-edible part of fruits can be used as natural antioxidants that might be used as phytomedicine. This is due to its function as secondary metabolites in plants defense system.
